# Post-transcriptional regulation of ITGB6 protein levels in damaged skeletal muscle

**DOI:** 10.1007/s10735-014-9567-2

**Published:** 2014-02-02

**Authors:** Melissa Ducceschi, Lisa G. Clifton, Stephen A. Stimpson, Andrew N. Billin

**Affiliations:** 1 Target and Pathway Validation, Molecular Discovery Research, GlaxoSmithKline, Research Triangle Park, NC USA; 2 Muscle Metabolism Discovery Performance Unit, Metabolic Pathways and Cardiovascular Therapeutic Area, GlaxoSmithKline, Research Triangle Park, NC 27709 USA

**Keywords:** Integrin beta 6, TGFbeta, Skeletal muscle, Regeneration, Repair

## Abstract

We have identified integrin beta 6 (*Itgb6*) as a transcript highly enriched in skeletal muscle. This finding is unexpected because *Itgb6* is typically associated with epithelial expression domains in normal tissue. Further we find that ITGB6 protein expression in muscle is post-transcriptionally regulated. Uninjured muscle expresses *Itgb6* RNA but no ITGB6 protein is detectable. Muscle injury induces ITGB6 protein accumulation rapidly post-injury in myofibers adjacent to the site of injury. As regeneration of the injured muscle tissue progresses ITGB6 protein is found in newly formed fibers up to at least 15 days post-injury.

## Introduction

Skeletal muscle demonstrates marked plasticity in response to changes in physical loads via hypertrophy or atrophy and to injury with a robust regenerative response. The extracellular matrix (ECM) has been defined as “…the non-cellular component present within all tissues and organs, and provides not only essential physical scaffolding for the cellular constituents but also initiates crucial biochemical and biomechanical cues that are required for tissue morphogenesis, differentiation and homeostasis” (Frantz et al. 2010). The ECM of skeletal muscle is composed of the basement membrane that directly abuts and surrounds the muscle cells and the interstitial matrix. The basement membrane is anchored to the muscle cells via muscle cell expressed integrin receptors and the basement membrane proteins Dystroglycan, Laminin, and Collagens. These cell and matrix interactions play a key role in maintaining the integrity of the tissue as it contracts and thus facilitates mechanical action of the muscle while buffering muscle from mechanical damage (Carmignac and Durbeej 2012).

The importance of the ECM in muscle structure and function is underscored by the existence of numerous genetically inherited diseases that alter ECM function and impact skeletal muscle function. Select examples include mutations in the laminin alpha 2 chain cause congenital muscular dystrophy 1a, mutations in collagen VI chains *COL6A1*, *COL6A2*, and *COL6A3* that are associated with Ullrich congenital muscular dystrophy and Bethlem myopathy, and various mutations in the dystrophin gene (*DMD*) that cause Becker Muscular Dystrophy, Dilated Cardiomyopathy 3B, and Duchenne Muscular Dystrophy (Mercuri and Muntoni 2013).

TGFβ signaling plays an important role in muscle regeneration and homeostasis of the ECM. Indeed, fibrosis of the skeletal muscle is a feature of the advanced stages of many myopathies and dystrophies in humans (MacDonald and Cohn 2012). TGFb is known to suppress myogenesis by targeting myogenic transcription factors (Liu et al. 2001, 2004). In addition, TGFβ production by aged satellite cells (skeletal muscle stem cells) has been implicated as a causative factor in the reduced ability of aged mouse muscle to regenerate as effectively as young mouse muscle (Carlson et al. 2008a, b; Carlson and Conboy 2007). The processes that regulate the production of TGFβ in muscle are therefore of interest as potential targets for interventions to modulate fibrosis and promote muscle repair in the elderly, muscular dystrophies, and myopathies.

In many tissues the ECM plays a role in regulating the production of active TGFβ from the inactive latent TGFβ or latency associated protein (LAP). One mechanism that regulates the production of TGFβ is via the action of the integrin receptor alphaVbeta6 (aVb6). The aVb6 receptor can bind to the LAP and thereby increase the exposure of the LAP to proteolytic activities that then liberates active TGFβ. The αVβ6 integrin has been considered an epithelia restricted integrin receptor as it has only been found on the surface of particular epithelial cells (Breuss et al. 1993) thus its role in regulating the production of active TGFβ in muscle has not been considered.

Here we describe the expression of the ITGB6 receptor mRNA and protein in murine skeletal muscle. We find that while the RNA for *Itgb6* is expressed in skeletal muscle the protein is not detected until after injury to the muscle. Thus we have identified a previously unappreciated mechanism that regulates ITGB6 expression in a non-epithelial tissue. This further suggests that ITGB6 protein production may be conditionally regulated in other non-epithelial tissues and that the domains of its activities extend beyond just epithelial tissues.

## Results

**Fig. 1:**
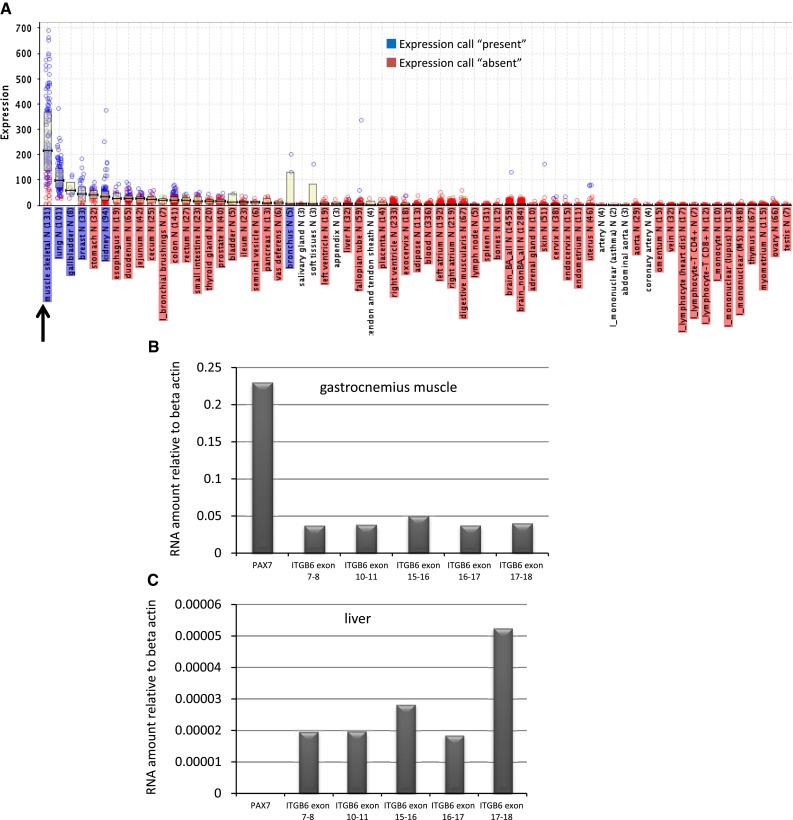
*Itgb6* RNA expression. **a** Plot of probe level intensity for Affymetrix probe 208084_at corresponding to the human ITGB6 transcript for a survey panel of tissues and cell types. The *arrow* denotes the skeletal muscle samples. **b** Taqman qPCR data from skeletal muscle RNA for multiple exons of *Itgb6* and *Pax7* (a positive control for muscle gene expression). **c** Taqman qPCR data from liver for multiple exons of *Itgb6* and *Pax7* (not detected in liver as expected)

TGFβ signaling is a major regulator of skeletal muscle regeneration. Regulation of the TGFβ signaling cascade in muscle may therefore be a useful method of therapeutic intervention for effective skeletal muscle regeneration. We thus set out to identify components of the TGFβ regulatory cascade by examining the GeneLogic database of human tissue microarray expression data for muscle expressed and enriched genes (Fig. 1a). This analysis identified genes such as *Myostatin*, a TGFβ family member known to be enriched in muscle as well as *Itgb6* (data not shown). This latter result was surprising given the studies of tissue distribution of *Itgb6* had not reported skeletal muscle *Itgb6* RNA expression (Breuss et al. 1993). However, multiple probes identified *Itgb6* as expressed in muscle as well as in other tissues that were previously documented as sites of *Itgb6* expression thus lending credence to the possibility that *Itgb6* is expressed in skeletal muscle.

We next determined whether *Itgb6* RNA is expressed in mouse skeletal muscle by quantitative real time PCR (qPCR). We tested 5 intron spanning primer/probe sets that detect exons 7 and 8, exons 10 and 11, exons 15 and 16, exons 16 and 17, or exons 17 and 18 of the NCBI RefSeq transcript NM_001159564.1. All 5 exon pairs were readily detected in skeletal muscle RNA but, as expected, were nearly absent from normal intact liver RNA (Fig. 1a, b). *Pax7* RNA was detected in skeletal muscle and was absent from liver RNA as expected.

**Fig. 2:**
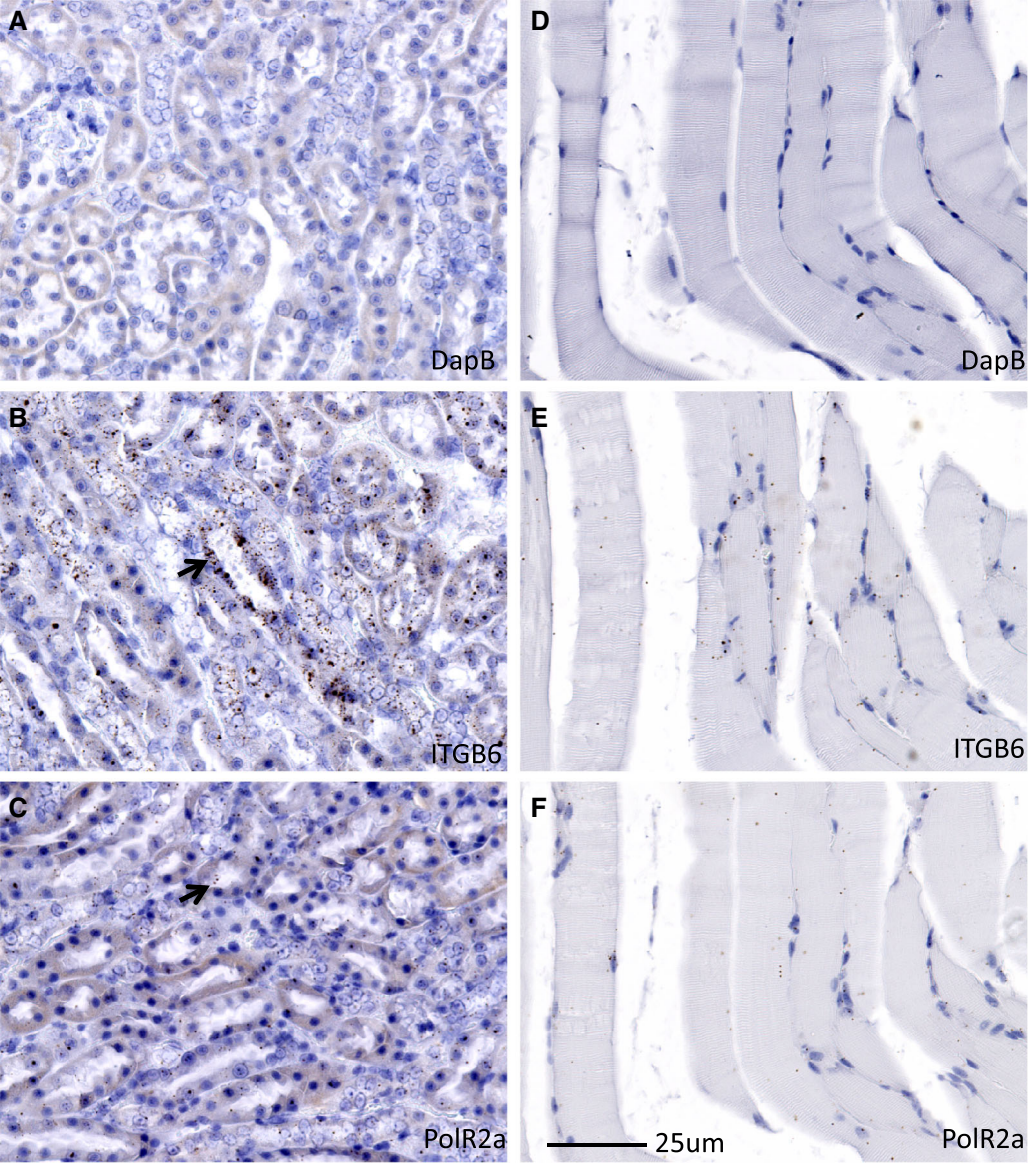
RNA expression by in situ hybridization. **a** Mouse kidney section hybridized with the negative control DapB probe shows no signal. **b** Kidney section hybridized with *Itgb6* probe shows abundant signal. **c** Kidney section hybridized with the positive control *PolR2A* shows expression of the transcript. **d** Muscle section hybridized with the negative control *DapB* probe shows no signal. **e** Muscle section hybridized with *Itgb6* demonstrates expression of the transcript. **f** Muscle section hybridized with the positive control probe for *PolR2a* shows expression

In addition to the myocytes themselves, skeletal muscle contains numerous other cell types that compose the vasculature, interstitial cells, and the synaptic termini of peripheral nervous system neurons. To determine the site of expression of *Itgb6* RNA in muscle tissue we performed in situ hybribization on mouse muscle samples. An in situ hybribization probe corresponding to nucleotides 533 to 1,496 of RefSeq transcript NM_001159564.1 was used in the RNAscope procedure. Mouse kidney tissue was first examined as a positive control for the *Itgb6* probe. Abundant signal was detected with the *Itgb6* probe in kidney tissue (Fig. 2b) whereas the negative control probe (*DapB*) detected nothing in the kidney sections (Fig. 2a). An additional positive control probe for the *PolR2a* gene also detected *Pol2Ra* transcripts in the kidney sections (Fig. 2c). The punctuate signal observed in these sections is typical of the signal observed with the RNAscope procedure. In sections of mouse skeletal muscle *Itgb6* RNA was detected and associated with myonuclei and myocytes (Fig. 2e) suggesting at least a portion of the muscle expressed *Itgb6* originates from myocytes per se. Again the negative control probe *DapB* demonstrated no staining (Fig. 2d) and the positive control probe *PolR2a* detected transcripts in the myocytes.

**Fig. 3:**
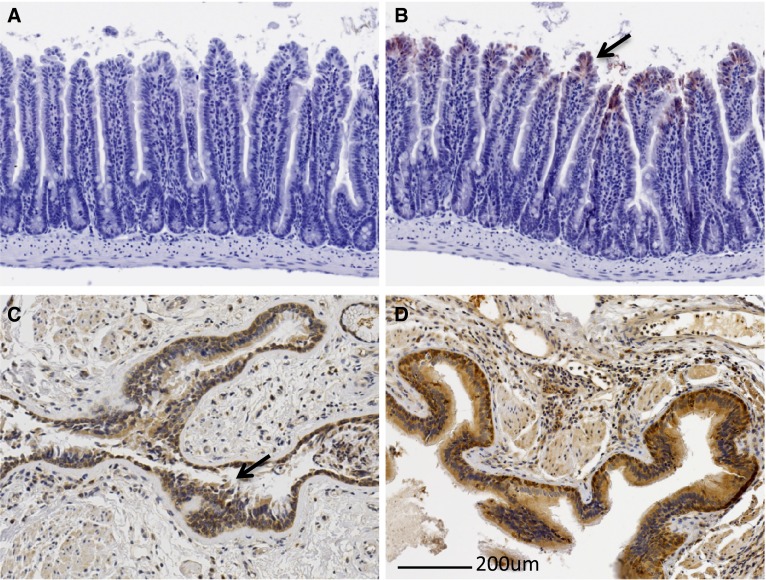
Antibody validation. **a** Mouse Ileum section stained with control IgG. **b** Mouse Ileum section stained with ITGB6 IgG. *Arrow* indicates stained epithelial region. **c** Normal human lung shows ITGB6 staining in the epithelial region (*arrow*). **d** Human emphysema lung demonstrates widespread ITGB6 staining

We next determined the localization of ITGB6 protein by immunohistochemistry (IHC) on sections of muscle tissue. First sections of tissue known to express ITGB6 protein were evaluated by IHC in order to validate the antibody. ITGB6 was detected in normal human lung, emphysema lung, and mouse ileum, all known sites of ITGB6 protein expression, whereas the control IgG sections had little or no signal (Fig. 3 a–d). We next examined mouse muscle sections for ITGB6 protein expression. Surprisingly we could not identify any sites of ITGB6 protein expression in muscle sections (not shown). This result suggested that despite widespread *Itgb6* RNA expression in muscle, no protein was being produced. We therefore hypothesized that *Itgb6* RNA may be under translational control in muscle and that an appropriate stimulus may be required to promote translation of the mRNA.

We used a freeze injury model to induce a localized area of tibealis anterior (TA) muscle tissue damage and then isolated damaged tissue at different times post injury and processed these for IHC of ITGB6. Serial sections were processed for control IgG and anti-ITGB6 staining.

**Fig. 4:**
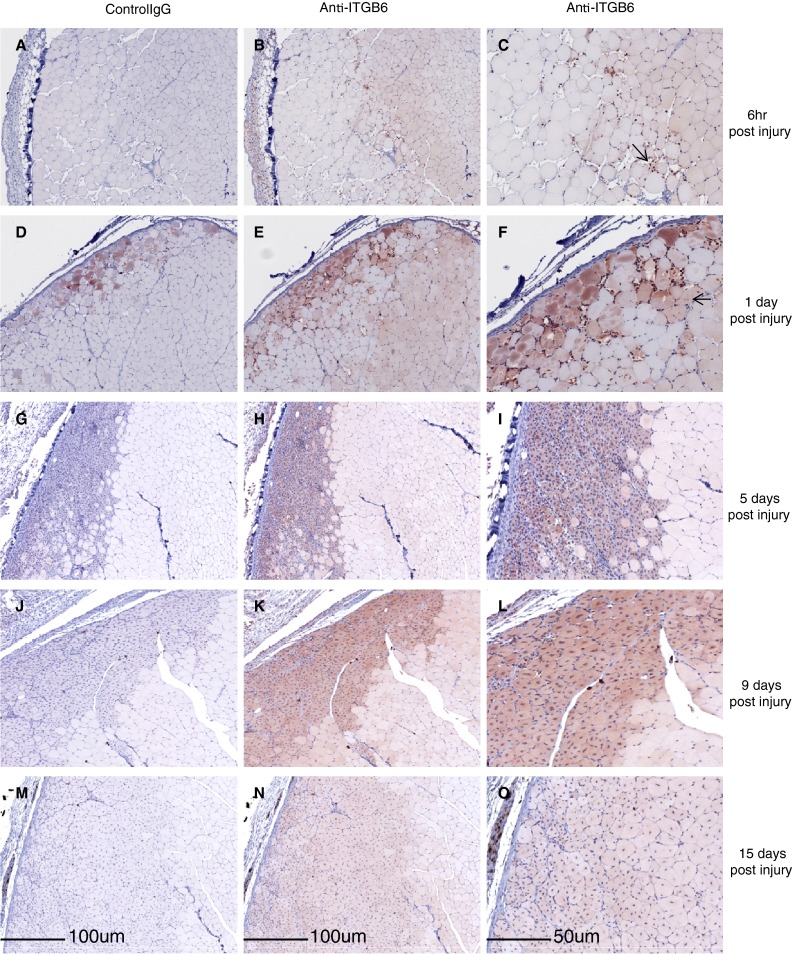
Mouse muscle post freeze injury time course. **a** 6 h post-injury control IgG. **b** 6 h post-injury. Light ITGB6 staining is detected in undamaged myocytes, **c** 6 h post-injury, magnified area of 4**b** showing intensely stained immune cells (*arrow*) **d** 1 day post-injury control IgG. **e** 1 day post-injury ITGB6 staining is detected in undamaged fibers. **f**. 1 day post-injury magnified area of 4**e**. ITGB6 staining is detected in some of the damaged fibers (*arrow*). **g** 5 days post-injury IgG control. **h** 5 days post-injury ITGB6 staining is detected in regenerating fibers (*boxed* region). **i** 5 days post-injury magnified area of 4**h**. **j** 9 days post-injury IgG control. **k** 9 days post-injury. Regenerated fibers still have centrally located nuclei and stain for ITGB6. (*boxed* region). **l** 9 days post-injury magnified area of 4**k**. **m** 15 days post-injury control IgG. **n** 15 days post-injury newly formed fibers with centrally located nuclei express low levels of ITGB6. **o** 15 days post-injury magnified area of 4**n**

At 6 h post injury swollen necrotic muscle fibers are observed below the dermis in the freeze injured muscle. At this time ITGB6 protein is detected in undamaged muscle fibers adjacent to the swollen necrotic fibers. Numerous invading cells (likely neutrophils) are intensely ITGB6 positive at this time point (Fig. 4a, b, c). By one day after injury (Fig. 4d, e, f) numerous ITGB6 positive immune cells persist in the injured muscle and muscle fibers in undamaged appearing areas continue to express ITGB6 protein. Fibers near the dermis also stain with the control IgG (compare Fig. 4d–f) however some dermal proximal myofibers appear to be negative in the control IgG section and positive when stained with anti-ITGB6 suggesting that some of the injured fibers express ITGB6 at this time. Between day one and day four post-injury myofibers continue to express ITGB6 in the injured and uninjured regions of the muscle. Five days post injury regenerating fibers, identified by centrally localized nuclei, express ITGB6 protein (Fig. 4g–i). At 9 days post-injury ITGB6 expression persists in regenerating fibers (Fig. 4j–l) and by day 15 post-injury ITGB6 protein is found at reduced levels in regenerating fibers.

**Fig. 5:**
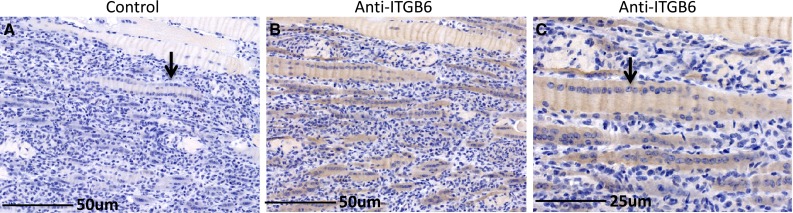
ITGB6 expression in *mdx* muscle. **a** Longitudinal section stained with control IgG. Note the nuclear “rowing” evident in the regenerating fibers (*arrow*). **b** ITGB6 is expressed in regenerating fibers with prominent nuclear “rowing”. **c** magnified area of 5**b**. *Arrow* indicates nuclear “rowing”

Freeze injury is not typically the type of injury that skeletal muscle is subject to and therefore the injury induced expression of ITGB6 could be peculiar to this model. We therefore examined ITGB6 expression in the muscles of the *Dystrophin* mutant *mdx* mouse, a genetic model of Duchenne Muscular Dystrophy. In this disease the muscle tissue is subject to continuous rounds of injury and repair due to the loss of *Dystrophin* and the resulting susceptibility of the muscle to mechanical damage. Examination of *mdx* muscle from 4 month old mice revealed the expected areas of regenerating muscle and associated with these areas expression of ITGB6 protein (Fig. 5). Thus, ITGB6 protein is expressed as a result of injury in two distinct models of muscle damage.

## Discussion

We have demonstrated that ITGB6 RNA is expressed in skeletal muscle and that translation of the RNA is not observed in normal uninjured muscle. Injury induces expression of ITGB6 in muscle fibers. Thus, our data implies the existence of post-transcriptional regulation of the ITGB6 gene at the level of RNA translation. This observation is novel for ITGB6 as this type of regulation has not, to our knowledge, been reported previously for ITGB6 messenger RNA. The signaling pathways regulating ITGB6 protein accumulation are not known, however this represents a potentially fruitful area for future research. The elucidation of the mechanism(s) may reveal novel injury induced signaling pathways in skeletal muscle that coordinate the elaboration of the regenerative process. ITGB6 is known to regulate the production of mature and biologically active TGFβ from the latent form of TGFβ and our data suggests that ITGB6 may also play such a role in injured skeletal muscle. Excessive TGFβ signaling is thought to contribute to detrimental changes in muscle functional properties in diseases like Duchenne Muscular Dystrophy and therefore regulation of ITGB6 activity may represent a fruitful area for drug discovery research.

## Materials and methods

### RNA extraction and quantitative real time PCR

RNA was extracted by Trizol, per manufacturer’s instructions. Real-time quantitative PCR analysis was performed using an ABI PRISM 7700 Sequence Detection System instrument and software (PE Applied Biosystems, Inc., Foster City, CA). Primers and probes were obtained from ABI.

### RNA in situ hybridization (ISH)

In situ hybridization for ITGB6 was performed using the RNAscope 2.0 FFPE Assay (Advanced Cell Diagnostics, Hayward, CA) according to the manufacturer’s directions. Briefly, 5 um formalin fixed paraffin embedded (FFPE) sections were pretreated with heat and protease before hybridization with the integrin beta 6 target probe. A horseradish peroxidase based signal amplification system was then hybridized to the target probe followed by visualization with 3, 3′-diaminobenzidine (DAB). Positive staining was observed as brown, punctate dots. Control probes for the bacterial gene DapB (negative control) and housekeeping gene PolR2a (positive control) were also performed for each tissue sample.

### Immunohistochemistry

Immunohistochemistry for integrin beta 6 was performed on 5um FFPE sections of human lung—which were sourced ethically and their research use was in accord with the terms of the informed consents—and on mouse ileum and muscle samples using an automated staining system (Dako Autostainer). Briefly, sections were deparaffinized and antigen retrieval was completed using Trilogy pretreatment solution (Cell Marque) in a pressure cooker for 20 min at 100  C. Sections were then incubated in 3 % hydrogen peroxide for 5 min to block endogenous peroxidase. The samples were then blocked using 10 % normal horse serum (Vector Laboratories) for 20 min followed by incubation with a goat anti-mouse integrin beta 6 polyclonal antibody (R&D Systems, catalog # AF2389) at 5ug/ml for 45 min at room temperature. Control non-immune goat IgG was used as a negative control to control for non-specific staining. The peroxidase ImmPRESS anti-goat polymer (Vector Laboratories) was applied for 30 min. Immunoreactive areas were visualized using the Romulin AEC chromogen kit (Biocare Medical).

### Animal care and models

All procedures performed were in compliance with the Animal Welfare Act and United States Department of Agriculture regulations and approved by the GlaxoSmithKline Institutional Animal Care and Use Committee. Mice were maintained on standard laboratory chow and allowed access to food and water ad libitum. Mice were C57Bl6/J or C57BL/10ScSn-*Dmd* ^*mdx*^/J and were obtained from Jackson Labs.

C57BL/6NTac male mice (Takonic), age 6 months, with an average body weight of 30.8 g, were used for the skeletal muscle freeze injury protocol. Briefly, mice were anesthetized with isoflurane and given a single subcutaneous injection of carprofen (3 mg/kg) to minimize pain during recovery from the procedure. Hair over the tibialis anterior (TA) muscle was removed and the skin was cleaned for aseptic surgery. A 1 cm incision in the skin was made to expose the thickest portion of the TA and the skin was gently retracted to expose the full width of the TA at its midpoint. At the TA midpoint, the flat surface at the end of a 4 mm round copper probe, pre-cooled in dry ice, was gently applied to the muscle for 5 s. This left a blanched well defined circle of frozen tissue that returned to muscle coloring as it thawed over the next few seconds. The incision was closed with a surgical clip. Mice were euthanized by thoracotomy under isoflurane anesthesia at the desired time points after application of the frozen probe. The left lower hind limb was removed by cutting above the knee and below the ankle to keep TA-associated tendons intact and preserve TA length, and the limb was fixed in 10 % buffered formalin. After fixation, the legs were decalcified and trimmed to expose the best injury profile prior to being embedded in paraffin for sectioning.
